# Intravascular Molecular Imaging: Near-Infrared Fluorescence as a New Frontier

**DOI:** 10.3389/fcvm.2020.587100

**Published:** 2020-11-23

**Authors:** Haitham Khraishah, Farouc A. Jaffer

**Affiliations:** ^1^Department of Medicine, Beth Israel Deaconess Medical Center and Harvard Medical School, Boston, MA, United States; ^2^Division of Cardiology, Cardiovascular Research Center and Harvard Medical School, Massachusetts General Hospital, Boston, MA, United States; ^3^Wellman Center for Photomedicine and Harvard Medical School, Massachusetts General Hospital, Boston, MA, United States

**Keywords:** intravascular imaging, atherosclerotic cardiovascular disease, optical coherence tomography, molecular imaging, near-infrared fluorescence (NIRF)

## Abstract

Despite exciting advances in structural intravascular imaging [intravascular ultrasound (IVUS) and optical coherence tomography (OCT)] that have enabled partial assessment of atheroma burden and high-risk features associated with acute coronary syndromes, structural-based imaging modalities alone do not comprehensively phenotype the complex pathobiology of atherosclerosis. Near-infrared fluorescence (NIRF) is an emerging molecular intravascular imaging modality that allows for *in vivo* visualization of pathobiological and cellular processes at atheroma plaque level, including inflammation, oxidative stress, and abnormal endothelial permeability. Established intravascular NIRF imaging targets include macrophages, cathepsin protease activity, oxidized low-density lipoprotein and abnormal endothelial permeability. Structural and molecular intravascular imaging provide complementary information about plaque microstructure and biology. For this reason, integrated hybrid catheters that combine NIRF-IVUS or NIRF-OCT have been developed to allow co-registration of morphological and molecular processes with a single pullback, as performed for standalone IVUS or OCT. NIRF imaging is approaching application in clinical practice. This will be accelerated by the use of FDA-approved indocyanine green (ICG), which illuminates lipid- and macrophage-rich zones of permeable atheroma. The ability to comprehensively phenotype coronary pathobiology in patients will enable a deeper understanding of plaque pathobiology, improve local and patient-based risk prediction, and usher in a new era of personalized therapy.

## Introduction

In 1989, Muller et al. (1) proposed the term “vulnerable plaque” to describe coronary artery plaques prone to rupture and cause acute myocardial infarction or sudden cardiac death. In 2003, a group of experts reached a consensus on a definition of vulnerable/high risk plaque being “a plaque that is at increased risk of thrombosis (or recurrent thrombosis) and rapid stenosis progression” (2). This definition extends beyond plaque rupture to include disruptions that may also occur from plaque erosions and penetrating calcified nodules. In general, the current paradigm of a high-risk plaque involves a thin cap fibroatheroma (TCFA), characterized by a large, necrotic, lipid-rich core and an overlying thin fibrous cap of <65 μm thickness. Other high risk features include inflammation, microcalcifications, neovascularization, intraplaque hemorrhage and positive remodeling (3–5).

High-resolution intravascular imaging modalities, such as intravascular ultrasound (IVUS) and optical coherence tomography (OCT), assess *in vivo* atheroma burden and some high-risk features reflecting plaque vulnerability (6). Despite these advances, current anatomical intracoronary imaging modalities have their shortcomings in predicting future acute coronary syndrome (ACS) events, even when incorporating advanced IVUS-derived high risk plaque measures, such as high plaque burden >70%, minimal luminal area <4 mm^2^, IVUS-virtual histology demarcated TCFA, and low endothelial shear stress (7–9). To address the limitations of anatomical intracoronary imaging, near-infrared fluorescence (NIRF) molecular imaging has emerged as a translational intravascular modality that allows visualization of plaque pathobiology by targeting certain molecular processes through specialized imaging agents [fluorophore-conjugates (10, 11) or protease-activatable constructs (12, 13) for example]. No single modality allows for the comprehensive evaluation of a plaque; to meet this need, hybrid catheters that allow co-registration of images acquired by different modalities have been developed. The aim of this review is to showcase the latest developments in molecular intravascular imaging, with a focus NIRF hybrid imaging modalities, and delve into current limitations and future prospects.

## Plaque Pathobiology

**Figure 1:**
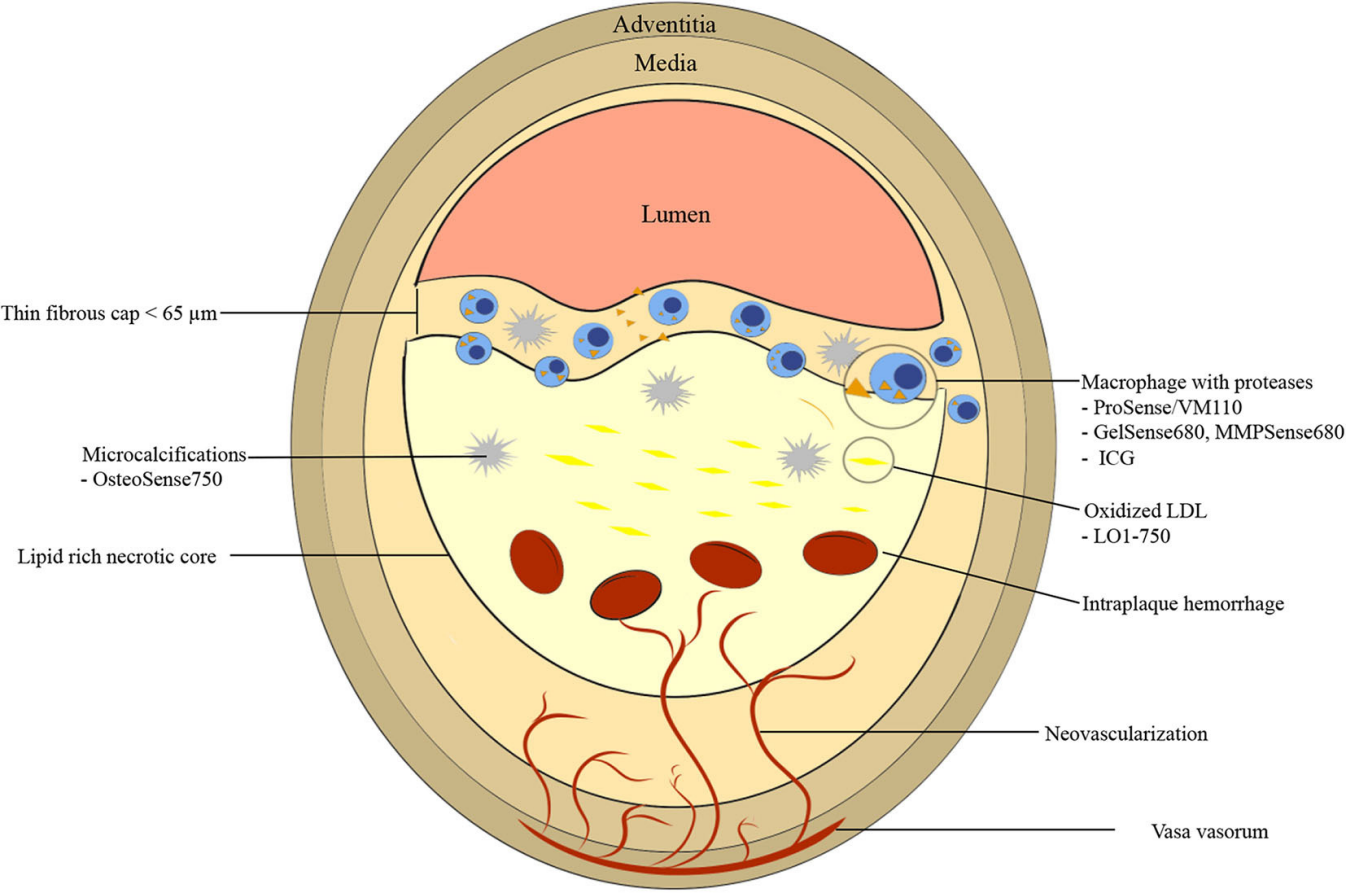
Graphic representation of vulnerable plaque characteristics with emphasis on near-infrared fluorescence (NIRF) molecular imaging targets. High risk features include thin fibrous cap <65 μm, fibrous cap and necrotic core macrophages, large necrotic core, microcalcifications, neovascularization, and intraplaque hemorrhage. ICG, indocyanine green.

The underlying pathobiology of atherosclerosis provides a roadmap for molecular imaging targets and imaging agent synthesis (Figure 1). Atherosclerosis is a chronic inflammatory disease characterized by endothelial dysfunction, arterial wall thickening and persistent immune activation. An initial step in atherogenesis is vascular endothelial dysfunction, triggered by an increase in oxidative stress within a milieu of hyperlipidemia, hyperglycemia, smoking and hypertension (14). Small low-density lipoprotein (LDL) particles infiltrate through the impaired endothelial barrier and accumulate in the subendothelial matrix (15, 16). Oxidized LDL (oxLDL) is internalized by local macrophages giving rise to foam cells (17–19). Vascular smooth muscle cells (VSMCs) migrate from the tunica media to intima in response to inflammatory mediators, growth factors, and cytokines. There, they proliferate and secrete extracellular matrix including proteoglycans and collagen type 2, contributing to plaque stability through the formation of a fibrous cap (20, 21). Apoptosis and necrosis of activated macrophages and VSMCs results in the formation of a lipid-rich necrotic core that is encapsulated by collagen-rich fibrous tissue, a fibrous cap (18, 22). When a fibroatheroma exists under a thin fibrous cap (thinned by inflammatory proteases such as MMPs and cathepsins), these advanced lesions are denoted as TCFAs, the current paradigm of high-risk plaque.

Another high-risk plaque feature is microcalcification. Calcification is an active process that occurs during all stages of atherosclerosis. Macrocalcifications, which are detectable by CT scan due to high radiodensity [>400 Hounsfield unit (HU)], may be associated with more stable plaque phenotypes (23). In contrast, microcalcifications (<0.05 to 15 μm) are undetectable by conventional imaging modalities and may have a destabilizing effect on fibroatheromas (24). When microcalcifications, arise within the fibrous cap, local stress increases by 2-fold, putatively contributing to plaque vulnerability (25). Microcalcification-aggregation results in macrocalcifications (18). Another high-risk vulnerable plaque feature is spotty calcifications, small calcium deposits that can be detected via OCT, IVUS, and CT. For IVUS and OCT, spotty calcification is defined as a calcium length <4 mm and a maximal calcium angle ranging from 22.5 to 90° (26). For CT, spotty calcifications are typically defined as average density >130 HU, diameter <3 mm in any direction, length of the calcium <1.5× the vessel diameter, and width of the calcification less than two-thirds of the vessel diameter (27).

Positive remodeling is thought to contribute to plaque instability by induction of neovascularization of vasa vasorum in the adventitia that eventually invade the intima and the fibroatheroma (28). These new microvessels have fragile walls leading to intraplaque hemorrhage (resulting in plaque expansion and pro-inflammatory milieu), and express adhesion molecules recruiting further macrophages into the plaque (Figure 1) (29).

## Detecting High Risk Plaque: Significance, Challenges, and Intravascular Imaging

The goal of detecting high-risk plaques is to identify rupture-prone plaques and intervene in a way (either medically or interventionally or both) to prevent future ACS. Very recently, the COMPLETE trial showed that in patients with STEMI and multivessel coronary artery disease (CAD), intervening on non-culprit lesions reduced a composite of CVD death and myocardial infarction (30), demonstrating the potential to identify a subgroup of high-risk, non-culprit lesions (admittedly a proportion of these lesions may have been flow-limiting and met common indications for revascularization, independently of the COMPLETE trial). However, in other clinical scenarios such as stable CAD, several challenges exist as to which non-culprit lesions to intervene upon. These challenges are due to imprecise knowledge of the natural history of plaque progression based on structural-based imaging trials and autopsy studies. Also, coronary artery plaques are of dynamic nature with lesion morphology oscillating from high risk to more stable and vice versa over time, as demonstrated by small longitudinal imaging studies (31–33). Additionally, current anatomical intravascular imaging modalities have limited ability to predict ACS arising from high risk plaques. In the PROSPECT trial, after incorporating all IVUS-derived predictive variables [high plaque burden >70%, minimal luminal area <4 square mm, or IVUS-virtual histology demarcated thin capped fibroatheroma (TCFA)], IVUS was limited in forecasting event-causing lesions with a relatively low positive predictive value (PPV) of 18.2% (meaning 4 out of 5 three-feature plaques did not produce in an event at 3 years) (9). Integrating endothelial shear stress parameters in the PREDICTION trial enhanced PPV to 41% (7), which is an improvement but still insufficient for routine clinical management. Therefore, new imaging approaches to more comprehensively phenotype atheroma are needed to improve lesion-specific risk prediction.

## Intravascular Molecular Imaging

To tackle the limitations of anatomical intravascular imaging modalities, intravascular molecular imaging has been developed to allow for *in vivo* visualization of molecular process that contribute to plaque vulnerability. The primary intravascular molecular imaging modality nearing clinical translation utilizes near infrared fluorescence (NIRF) detection of targeted NIR fluorophores, discussed in detail next.

## Near-Infrared Fluorescence (NIRF)

### Basics of Near-Infrared Fluorescence (NIRF) Molecular Imaging

Near-infrared fluorescence (NIRF) is an emerging translational, intravascular imaging modality that has the ability to capture a wide range of *in vivo* pathobiological processes (34). NIRF molecular imaging entails (1) injecting targeted or activatable NIRF molecular imaging agents, which consist of fluorescent conjugates (e.g., NIR fluorophores conjugated to an antibody, peptide, or small molecule) that concentrate in atheroma and bind to molecular targets, and (2) detecting the fluorophore emission signal from a NIRF catheter and console detection system (35). After injecting targeted fluorophores, excitation light from the near-infrared spectrum (650–900 nm) is directed at the arterial wall and used to stimulate fluorophores from ground state (S0) to an excited state (S1, S2). The excited fluorophores (S1, S2) emit energy in the form of photons (fluorescence emission) and then return to the ground state, and are then available for further excitation. Fluorescence emission occurs at a lower energy and a longer wavelength, and this emission light is detectable with a high sensitivity charge-coupled device (CCD) camera and appropriate emission filter that attenuates the initial shorter wavelength excitation light (34). Compared to visible light range fluorescence detection, characteristics of NIRF imaging that make it a highly sensitive imaging modality include: (A) less light absorption by hemoglobin, lipid, and water, allowing deeper penetration of light into tissue; and (B) reduced background tissue autofluorescence, allowing for high signal-to-background ratio (36, 37).

**Figure 2:**
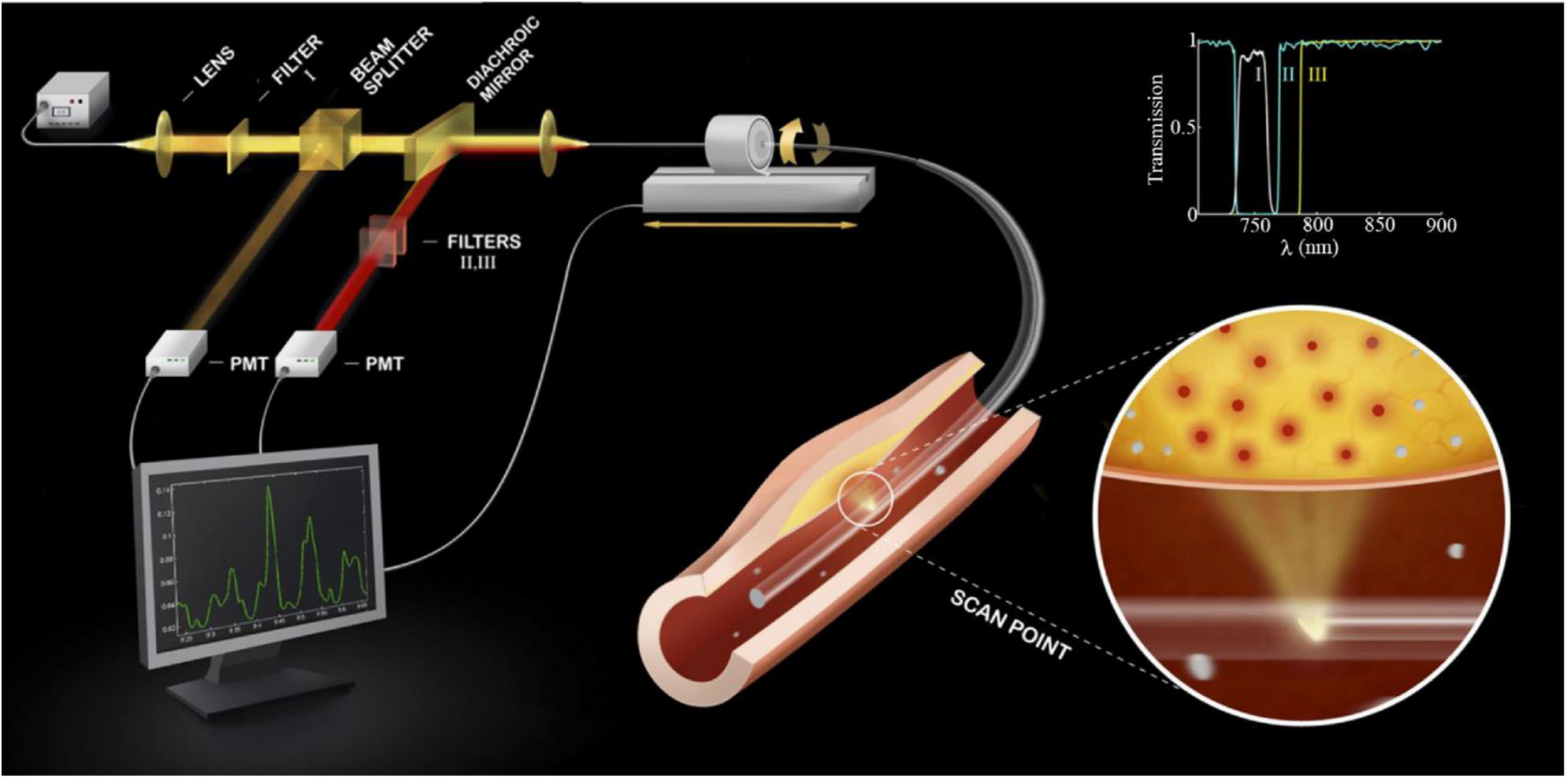
Diagram of a first-generation standalone 2D NIRF Imaging System. The tip of the probe contains a right angle coated prism that focuses laser light into the artery wall, activating fluorophores to their excited state to allow fluorescence emission. Subsequently, fluorophores will emit longer wavelength (lower energy) fluorescent light back into the optical fiber. The fluorescent light is then directed to a dichroic beam splitter that selectively directs it into a photomultiplier tube. The beam passes additional filters to minimize the parasitic signals of laser photons and autofluorescence. The inset shows the spectra of the three filters (I, II, III) used in the system. 2D, 2-dimensional; NIRF, near-infrared fluorescence. Jaffer et al. (13), by permission of American College of Cardiology. License number: 4720261188070.

In 2008, Jaffer et al. (38) described the first intravascular, real-time NIRF catheter-based spectroscopic sensing probe (Figure 2). After intravenous injection of ProSense750/VM110, a cathepsin protease-activatable NIRF agent, it was possible to detect *in vivo* plaque inflammation in rabbit iliac arteries (1.5–2.5 mm in diameter) through blood (38). This system was able to detect NIRF signal in spectroscopic-type mode from a limited section of the circumferential arterial wall, given its non-rotational nature. As a result, in 2011 our group developed a two-dimensional rotational NIRF intravascular catheter with automated pullback, providing real-time, *in vivo* spatial mapping of arterial inflammation in atherosclerosis and in stented rabbit aortas (13). It is noteworthy that molecular and anatomical intravascular imaging provide complementary information about plaque structure and biology. This motivated the development of multimodal NIRF hybrid catheter systems that combines NIRF with either OCT or IVUS, for both molecular-structural co-registration, and improved quantification of NIR fluorescence signals.

### NIRF Molecular Imaging Agents for Atheroma Targets

A number of NIRF molecular imaging fluorophores that target different molecular processes are now available. Those processes include protease activity, oxidized LDL, endothelial permeability, fibrin deposition, and microcalcifications. Neovascularization can be potentially detected using αv β3 integrin (39); however, to date it has not been studied for atheroma detection using intravascular NIRF catheters. At present, indocyanine green (ICG) is the only fluorophore approved by the US Food and Drug Administration (FDA) for use in human subjects, although a number of fluorophores are undergoing FDA approval in the oncology domain (40–43) and are expected to be translated to the field of atherosclerotic cardiovascular disease (CVD).

### Inflammatory Protease Activity

Atheroma macrophage inflammatory activity can be detected using protease-activatable NIR fluorophores. Cathepsins and matrix metalloproteinases (MMP) are amongst the most widely studied proteases. Cysteine cathepsins are mainly found in lysosomes and have been in implicated in atheroma-blood vessel remodeling through elastolytic and collagenolytic activity (44–46). ProSense/VM110 is a cathepsin-activatable fluorophore that has been validated in animal models of atherosclerosis, and very recently has been evaluated in a patient (47). Once injected, baseline quenched ProSense/VM110 localizes to atheroma macrophages, where it is cleaved by cathepsins, yielding NIR fluorescent fragments (48). Two NIR versions of ProSense exist: ProSense 680, which is activated by Cathepsin B, L, and S with peak excitation of 680 nm and peak emission of 700 nm, and ProSense 750/VM110, activated by Cathepsins B, L, S, K, V, and D with peak excitation of 750 nm and peak emission of 780 nm (48). MMPs are another group of proteases that have been implicated in plaque destabilization (49). Similar to ProSense, gelatinases (GelSense680 and MMPSense680) are MMP-activatable fluorophores that exhibit a mosaic distribution of NIRF signal, differentiating hot spots, which correlate with plaque instability (50, 51). Although label-free OCT detection of macrophages is possible (52, 53), NIRF offers a unique capability to visualize macrophage inflammatory activity *in vivo* is NIRF. A limitation is the need to inject ProSense/VM110 24 h prior to intended imaging; new inflammation sensors with faster pharmacokinetics are needed.

### Oxidized Low-Density Lipoprotein

Oxidized LDL (oxLDL) particles are often found within atheroma lipid-rich necrotic core and have been explored as targets for NIRF imaging. Khamis et al. (11) designed a oxidized LDL NIRF targeted molecular imaging agent, termed LO1-750, that binds to oxLDL and fluoresces when illuminated with 750 nm light. LO1-750 has two components: LO1, which is an autoantibody that binds to oxidized LDL in mice, rabbits and humans (54), and AF750, which is a NIRF dye. By utilizing fluorescence molecular tomography (FMT) combined with micro-computed tomography (CT), the group showed LO1-750 accumulation within the aortic arch and its branches in atherosclerotic Ldlr ^−/−^ mice when compared to wild type (WT) (11). LO1-750 generated higher NIRF signal when compared to MMPSense signal. *Ex vivo* imaging of rabbit aortas using intravascular NIRF catheter, showed localization of LO1-750 in atheroma lesions. This agent represents a translatable platform for future use in human subjects to enable quantifying plaque oxidative stress. A limitation for LO1-750 long circulating half-life (21 h) and wide area of distribution to the liver, kidneys, and spleen, limiting the plaque target-to-background ratio (11).

### Inflammation Determined by Impaired Endothelial Permeability

**Figure 3:**
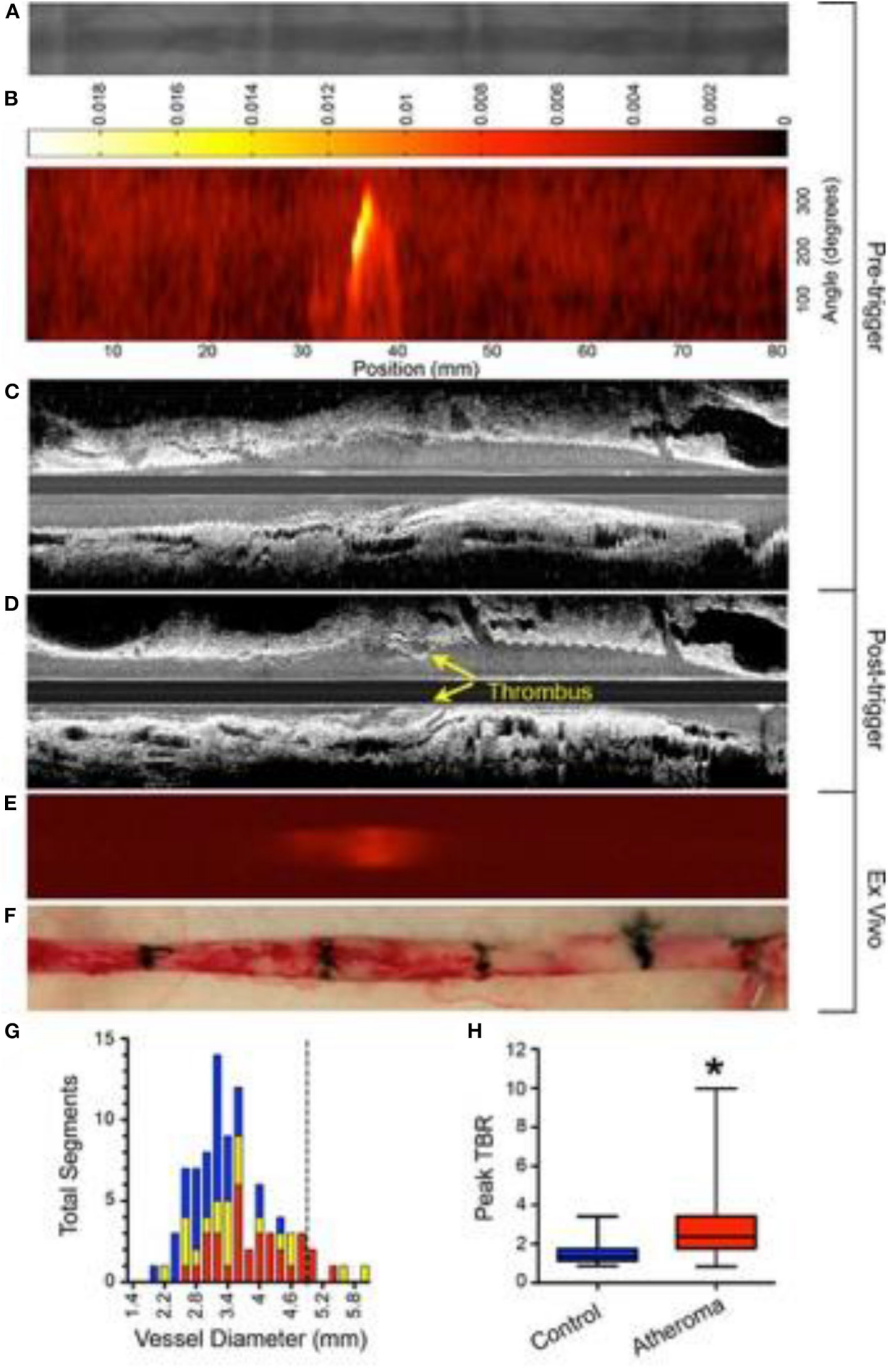
Experimental *in vivo* and *ex vivo* near-infrared fluorescence (NIRF) imaging of inflammation and subsequent atherothrombosis. Rabbits underwent balloon injury at week 2, and were fed high cholesterol diet till week 8. Ten weeks after balloon injury, rabbits received CLIO-CyAm7 (a inflammation-sensitive nanoparticle-based fluorophore), and underwent pharmacologic-triggered plaque thrombosis 24 h later. **(A)** Pre-trigger x-ray angiography showing the aorta for image coregistration. **(B)** Pre-trigger *in vivo* NIRF imaging projected into a 2-dimensional (2D) matrix showing an area of increased signal between 30 and 40 mm. **(C,D)** Pre- and post-trigger intravascular ultrasound (IVUS) imaging demonistrating induced luminal thrombus (yellow arrows) corresponding to the region of increased NIRF signal intensity on pretrigger NIRF imaging in **(B)**. **(E,F)** *Ex vivo* fluorescence reflectance imaging of cross-linked iron oxide (CLIO)-CyAm7 verifying *in vivo* 2D NIRF imaging, and gross pathology of the resected aorta with 1.5 cm black tissue markings for histological analysis and coregistration. **(G)** Histogram of vessel diameter measured by cross-sectional IVUS imaging. Red indicates atheroma without attached thrombus, yellow indicates atheroma with attached thrombus, and blue indicates uninjured control aortic segments. The dashed line indicates the 5 mm cut-off for exclusion of NIRF imaging data because of distance attenuation of the NIRF signal in large vessels. **(H)** *In vivo* 2D NIRF imaging revealed significantly higher target/background ratio (TBR) in areas with atheroma, compared with uninjured segments of the aorta (peak TBR 2.86 ± 1.82 and 1.55 ± 0.65, *P = 0.001). Stein-Merlob et al. (55), by permission of American Heart Asssociation (AHA). License number: 4720560395030.

Plaques that demonstrate impaired endothelial permeability are prone to erosion and thrombosis. Utilizing iron oxide nanoparticles (CLIO), Stein-Merlob et al. (55) developed a NIRF fluorophore, called CLIO-CyAm7 that was used to investigate *in vivo* endothelial dysfunction-based inflammation in a rabbit model. The group demonstrated that within atheroma plaque, CLIO-CyAm7 deposited in macrophages and endothelial cells in areas of impaired endothelial barrier underlying areas of triggered plaque thrombosis (Figure 3) (55). This insight links surface inflammation and endothelial permeability to plaque rupture *in vivo*. A limitation of this approach is the use of a long circulating nanoparticle, which may limit point-of-care applications in the cath lab.

### Fibrin Deposition

Subclinical plaque rupture may result in fibrin deposition at the plaque surface. In addition, unhealed stents are characterized by fibrin deposition and absent endothelium, and are at increased risk of stent thrombosis (56). In 2012, Hara et al. (10) developed FTP11-Cy7, a fibrin-targeted peptide (FTP11), conjugated to a NIRF dye (Cy7). FTP11-Cy7 binding to fibrin was validated *in vitro* using human plasma clots and *in vivo* by binding to murine thrombi using non-invasive NIRF imaging (10). Subsequently, using rabbit model and hybrid NIRF-OCT intravascular imaging, our group was able to demonstrate that drug eluting stents (DES) showed increased fibrin deposition and fibrin persistence when compared to bare metal stents (BMS), both at day 7 and day 28 (57). This finding also revealed the limitations of standalone OCT imaging, which cannot distinguish between healthy endothelial cell coverage vs. impaired healing demarcated by fibrin deposition. Indeed, a considerable percentage of stent struts appeared covered on OCT; however, a substantial portion of OCT covered struts were in fact covered by NIRF+ fibrin, rather than re-endothelialization, and therefore may indicate an increased risk of thrombosis. A possible limitation is NIRF imaging is not possible beneath the metallic stent struts.

### Microcalcifications

Similar to the process of bone formation, plaque calcification is a tightly regulated process of mineralization through the differentiation of VSMCs into osteoblasts that express osteogenic regulating proteins such as alkaline phosphatase, osteopontin, osteocalcin, osteonectin and collagen types I and II (58). When microcalcifications arise within the fibrous cap, local stress increases by 2-fold, contributing to plaque vulnerability (25). A seminal NIRF imaging study in mice demonstrated the ability to detect osteogeneic activity, which precedes bulk calcification, in calcifying murine aortic valves (59). Vulnerable plaques are characterized by microcalcifications and osteogenic activity, both of which are associated with macrophage burden. OsteoSense750 (excitation 750 nm) is a NIRF agent that is derived from bisphosphonate and binds to sites of calcification *in vivo*, highlighting osteoblastic activity, and hence, potential target for vulnerability (58, 60–62). Microcalcifications can be detected by OCT; however, OCT has difficulty in visualizing cellular-level calcifications. Also NIRF has the ability to visualize osteoblastic activity (63). Only a limited studies are available on the use of OsteoSense in vascular biology and plaque vulnerability (59).

## Hybrid Intravascular Near-Infrared Fluorescence Molecular Imaging

Standalone NIRF imaging does not allow for distance correction and localization of the source of NIR light excitation and fluorescence emission. In addition, separate NIRF catheter-based imaging and then structural based imaging (e.g., IVUS, OCT) is impractical for clinical use. For these reasons, hybrid intravascular molecular-structural catheters have been developed to enable concurrent co-registration of molecular and anatomical images, and accurate distance-based correction/quantification of the NIRF signal (Tables 1, 2) (64, 66).

**Table 1:** Individual and hybrid intravascular imaging in terms of detecting high risk plaque features.

	**NIRF**	**OCT**	**IVUS**	**NIRF-OCT**	**NIRF-IVUS**	**NIRF-OCT-IVUS**
Thin fibrous cap <65 μm	–	++	–	++	–	++
Lipid core	+ + + (LO1-750)	+ + +	+	+ + +	+ + +	+ + +
Microcalcifications	+ + + (OsteoSense)	+	+	+ + +	+ + +	+ + +
Macrophage infiltration	+ + +(ProSense, LUM015, MMPSense, and GelSense and ICG)	++	–	+ + +	+ + +	+ + +
Neovascularization	+Bevacizumab-IR800, Integrisense	+	–	++	+	++
Remodeling	–	–	++	–	++	++

*IVUS, intravenous ultrasound; NIRF, near-infrared fluorescence; OCT, optical coherence tomography*.

**Table 2:** Characteristics of NIRF hybrid intravascular molecular imaging.

		**Probe**	**Fiber**	**Depth of**	**Axial**	**Frame**	
		**diameter**	**characteristics**	**penetration**	**resolution**	**rate**	**Limitation**
NIRF-OCT	Hara et al. (57)	2.4 Fr	• OCT: 1320 nm • NIRF: 750 nm	NIRF: 3 mm	OCT: 7 μm	25.4/s	Slower image acquisition speed
	Zaheer et al. (60)	2.6 Fr	• OCT: 1,290 nm • NIRF: 749–790 nm	OCT: 1–2 mm	OCT: 7 μm	100/s	–
NIRF-IVUS	Shi et al. (63)	4.2 Fr	• IVUS: 45 MHz • NIRF: 750 nm	• IVUS: 4 mm • NIRF: 2 mm	IVUS: N/A	30/s	Large probe size
	Abran et al. (64) (Coronary)	4.5 Fr	• IVUS: 40 MHz • NIRF: 750 nm	• IVUS: 4 mm • NIRF: 2 mm	IVUS: 150 μm	2.7/s	Large probe size
	Abran et al. (64) (Peripheral)	9.0 Fr	• IVUS: 15 MHz• NIRF: 750 nm	• IVUS: 4 mm • NIRF: 2 mm	IVUS: 270 μm	2.7/s	Large probe size
NIRF-IVUS-OCT	Maehara et al. (65)	3.9 Fr	• OCT: 1,310 nm • IVUS: 40 MHz • NIRF: 785 nm	• OCT: 3–5 mm • IVUS: 5–6 mm NIRF: 3–5 mm	–	20/s	Large probe size

*IVUS, intravenous ultrasound; NIRF, near-infrared fluorescence; OCT, optical coherence tomography*.

### Hybrid NIRF-OCT

**Figure 4:**
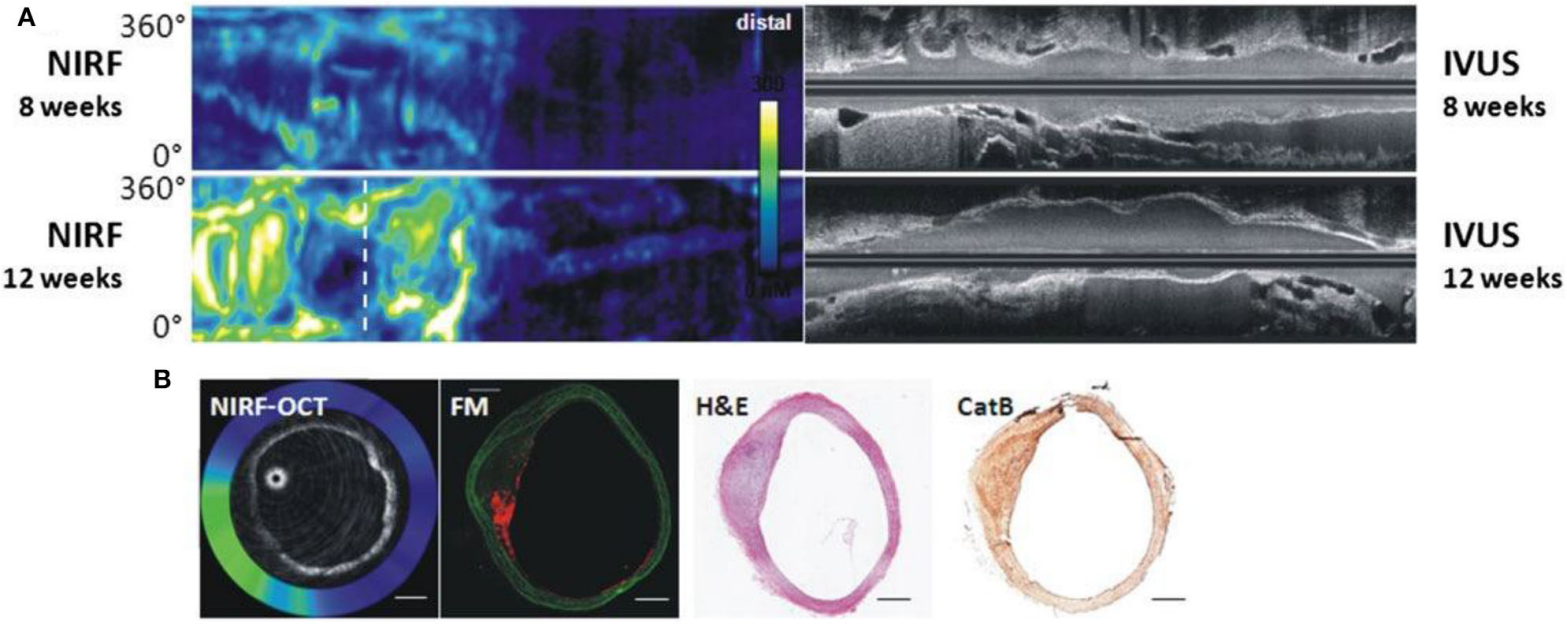
Dual modality NIRF-OCT along separate IVUS images, obtained at weeks 8 and 12 following balloon injury in rabbit aorta. ProSenseVM110 is the fluorophore used to acquire NIRF imaging. **(A)** Longitudinal rabbit aorta sections. To the left, co-registered OCT-NIRF images and to the right corresponding IVUS images. **(B)** Cross sections of rabbit aortas corresponding to white dotted line in **(A)**. From left to right: cross-sectional co-registration of NIRF-OCT images (Green/white = high near-infrared fluorescence; blue/black = low near-infrared fluorescence), matched cross-sectional fluorescence microscopy (FM) (red = ProSense VM110; green = autofluorescence) and histopathological sections reveals increased ProSense VM110 NIRF signal within a moderate fibrofatty atheroma (H&E) associated with cathepsin B immunostain. Scale bars, 1 mm. Figure courtesy of Dr. Eric Osborn and Dr. Giovanni Ughi. Bourantas et al. (67), by permission of European Society of Cardiology. License Number: 4720391246688.

OCT generates images with high spatial resolution (10–20 μm) but with relatively low depth of penetration (1–2 mm) (65). When combined with NIRF, the resulting hybrid catheter provides complementary data on plaque structure and biology (Figure 4). Two hybrid NIRF-OCT catheters have been developed; due to the OCT component, saline/contrast flushing is required during imaging acquisition. In 2011, Yoo et al. (66) developed a dual modality NIRF-OCT rotary catheter system with automated pullback and probe size of 2.4 Fr. The imaging probe comprised of a double-clad fiber with a single mode OCT core (1,320 nm) and inner cladding for NIR fluorescence collection (66). The system was validated using cadaveric human coronary artery with an implanted NIRF-fibrin labeled stent, and *in vivo* rabbit iliac arteries after balloon injury (66). Similarly, Lee et al. (68) designed a 2.6 Fr NIRF-OCT probe with frame acquisition rate of 100/s compared to 25.4/s in the Yoo et al. first-generation system. Using atherosclerosis rabbit models, ICG deposition was reliably identified in lipid- and macrophage-rich plaques *in vivo* (68). Later, the same group validated the use of NIRF-OCT system in drug eluting stent (DES)-stented swine coronary arteries, showing ICG localization within atheroma and behind implanted DES (69). Since NIRF-OCT is an all-optical fiber-based system, the engineering of the probe is more straightforward, compared to NIRF-IVUS or NIRF-IVUS-photoacoustic systems. Limitations of this system are low depth of penetration, and the need to flush during OCT image acquisition.

### Hybrid NIRF-IVUS

IVUS was introduced to clinical practice in early 1990s (70), and ever since has been the most widely used intravascular imaging modality due to decent depth of penetration (5–8 mm) and moderate backscatter from blood between 20 and 50 MHz (does not require blood clearance), with a limitation of intermediate spatial resolution (100–250 μm) (65). Dixon and Hossack (71) designed the first NIRF-IVUS hybrid probe and validated its use in phantom coronary arteries. One limitation of this system was the relatively larger probe size of 4.2 Fr due to side-by-side probe arrangement, precluding its use in typical diameter coronary arteries. Subsequently, Abran et al. (64, 72) developed a similar NIRF-IVUS bimodal catheter, and assessed its validity using ICG in *ex vivo* rabbit atherosclerosis model. The first *in vivo* validation of a bimodal NIRF-IVUS system was demonstrated by Bozhko et al. (73) in angioplasty-induced vascular injury in swine peripheral arteries and experimental fibrin deposition on coronary artery stents, and of atheroma in a rabbit aorta, using ICG. Clinical translation of this hybrid catheter necessitates designing a smaller probe <3.0 Fr, appropriate for intracoronary use, and will likely require re-engineering of side-by-side designs to serial designs, similar to current clinical IVUS-NIR spectroscopy catheters.

### Hybrid trimodal NIRF-OCT-IVUS Catheter

A tri-hybrid intravascular probe using IVUS, OCT and NIRF technologies was developed by Li et al. (74), offering the potential for multi-structural and molecular imaging in a single pullback. The system was validated using phantom and *ex vivo* experiments using pig and rabbit arteries (74). While this system offers the advantages of the three imaging modalities combined, the probe size at present (3.9 Fr) is currently too large for routine clinical type applications, but is an exciting advance nonetheless, and we await further validation *in vivo*.

## NIRAF-OCT Imaging

While NIRF intracoronary molecular imaging has not been performed in human subjects, a recent advance was the demonstration of clinical intracoronary OCT-NIR-autofluorescence (NIRAF, based at 633 nm), a new contrast-free autofluorescence-based method (75). In the first human study on 12 patients with CAD undergoing PCI, Ughi et al. (75) demonstrated that NIRAF signal correlated with vulnerable features like fibroatheroma, plaque rupture and in-stent restenosis in non-culprit lesions (75). Additionally, NIRAF signal was associated with components of components of intraplaque hemorrhage (e.g., bilirubin, protoporphyrin IX) (76). While NIRAF molecular imaging is very promising intravascular imaging modality, the complete set of molecules and biological processes underlying NIRAF remain to be elucidated. Compared to NIRAF, NIRF imaging through molecular-specific imaging agents allows targeting of specific patho-biological processes, like protease activity, oxidized LDL, endothelial permeability, fibrin deposition, and osteogenesis.

## Photoacoustic Imaging

Intravascular photoacoustic (IVPA) is an emerging imaging modality that is a natural extension of IVUS, and has the potential to detect certain fluorophores and nanomaterials that could enable IVPA-based molecular imaging. IVPA is a novel structural and molecular imaging modality that uses multiple wavelengths to illuminate the tissue of interest and identifies the acoustic waves generated by the thermoelastic expansion of the environment surrounding absorbing molecules (77). In an *in vivo* experiment on rabbit aortas, Wang et al. (78) showed that IVPA/IVUS imaging can identify lipid deposits within vessel wall through the blood without the need of saline flushing or balloon occlusion. Excitingly, IVPA provided 3 dimensional images of the vessel wall. Recently, Iskandder-Risk et al. demonstrated IVPA *in vivo* imaging of swine coronary artery. The quality of images were adjudicated using OCT and histology (79).

## Translational Outlook

**Figure 5:**
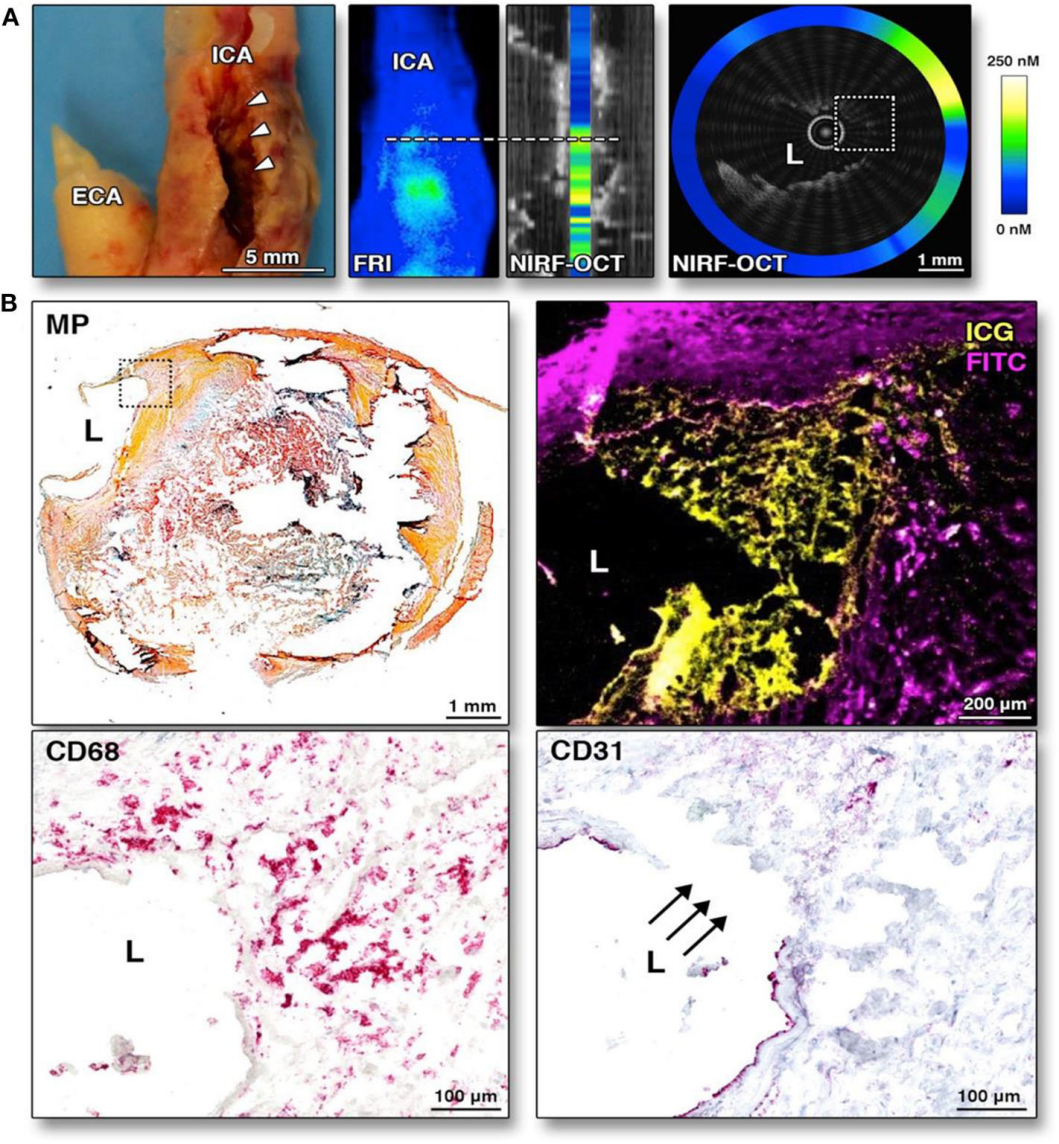
*Ex-vivo* intravascular NIRF-OCT images of human carotid arteries following ICG injection and then endartectomy. ICG was injected into human subjects 99 min prior to endartectomy. ICG accumulates in atherosclerotic area especially endothelial discontinuation. **(A)** Corresponding gross internal carotid artery (ICA) specimen, along with corresponding fluorescence reflectance image (FRI), and NIRF-OCT cross sectional, coregistered images, illustrating similar ICG uptake pattern (light blue = low ICG signal; green-yellow = high ICG signal) at the stenotic region (white arrowheads) in the ICA. **(B)** Histological analysis of the same area of the NIRF-OCT cross-sectional image shown in **(A)**. Movat pentachrome (MP) shows a complex atherosclerotic plaque with a large necrotic core with lipid and cellular infiltration (dotted box). Higher magnification (10×) fluorescence microscopy of the boxed area illustrates ICG NIRF signal adjacent to the lumen, which is distinct from fluorescein isothiocyanate (FITC)-channel autofluorescence. CD68 staining of the same area validates that the ICG NIRF signal (yellow pseudocolor) spatially relates to CD68-defined plaque macrophages beneath the area of intimal disruption. The disruption is confirmed by CD31 staining in this same area. ECA, external carotid artery. Verjans et al. (81), by permission of American College of Cardiology (ACC). License number: 4720551416574.

To enable clinical intracoronary NIRF molecular imaging, the NIRF system will need to switch to 750–800 nm excitation, and clinical targeted/activatable NIRF imaging agents will need to be available. Currently, indocyanine green (ICG, ex/em 805/830 nm) is the only atherosclerosis-targeted NIR fluorophore approved by the US Food and Drug Administration (FDA) for human use, facilitating the use of NIRF catheter in human subjects once approved. The recognition that ICG, a blood flow imaging agent available for 50 years, could target atherosclerosis was somewhat unexpected. In 2011, Vinegoni et al. recognized that the amphiphilic properties of ICG might allow it to have an atheroma targeting profile. Using intravascular and *ex vivo* NIRF imaging, ICG was found to accumulate in atheroma macrophages and was detected *in vivo* rabbit atheroma (80), and in pig coronary plaques (69, 81). Subsequently in 2016, The BRIGHT-CEA trial (Indocyanine Green Fluorescence Uptake in Human Carotid Artery Plaque, a study of injection of ICG prior to patients undergoing carotid endarterectomy) was conducted (81). Five patients undergoing carotid endarterectomy were injected with ICG; plaques were resected 99 min afterwards. *Ex vivo* intravascular NIRF-OCT and fluorescence reflectance imaging showed that ICG NIRF signal localized to and accumulated in human atheroma for the first time in living patients (Figure 5). The authors concluded that ICG accumulated in plaques with impaired endothelial integrity, disrupted fibrous cap, and area of neovascularization (81). This study may pave the way for future human intracoronary NIRF-OCT using ICG to image pathobiological aspects of coronary atherosclerosis.

**Figure 6:**
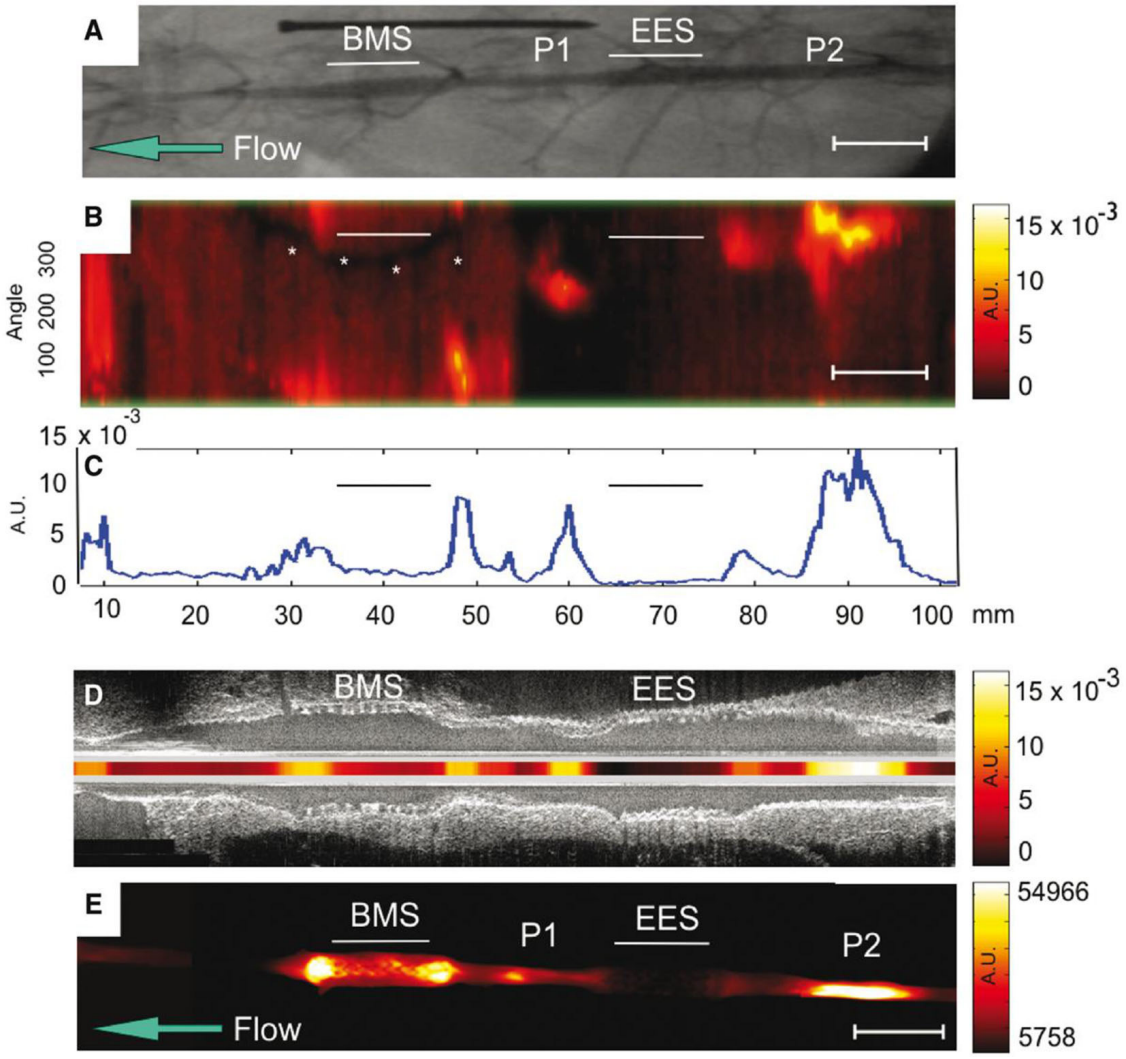
NIRF molecular imaging of stent suppresion of plaque inflammation. *In vivo* and *ex vivo* imaging of rabbit aorta demonestrating inflammatory protease activity in bare metal stent (BMS)-, everolimus-eluting stent (EES)-treated, and unstented plaque zones. **(A)** Angiogram of the abdominal aorta, showing positions of BMS and EES. Areas of IVUS–visible plaque areas (P1 and P2 zones) are highlighted. **(B)** *In vivo* NIRF imaging, demonestrating increased signal intensity in areas corresponding to BMS. The y-axis represents the angular dimension (0–360°). The x-axis represents the longitudinal/axial dimension in millimeters. The asterisk denotes a guidewire artifact. **(C)** Averaged mean NIRF signal (unidimentional) along the longitudinal axis. NIRF signal is higher in unstented regions > BMS regions > EES regions **(D)** Co-reistered longitudinal IVUS and intravascular NIRF images. **(E)** *Ex vivo* FRI at 800 nm of the resected aorta, demonestrating higher signal in BMS region, compared to EES region. AU, arbitrary units; Scale bar, 10 mm. Calfon Press et al. (12), by permission of European Society of Cardiology. License number: 4720551036244.

Another translatable aspect is the use of NIRF imaging to detect plaque inflammation modified by stent placement, and predict complications such as in-stent thrombosis and stenosis. Using an atheroma rabbit model, Calfon Press et al. (12) showed that everolimus-eluting DES could decrease *in vivo* plaque inflammation and macrophage accumulation. This finding provides evidence that DES may be a bio-stabilizer of high-risk, inflamed plaques (Figure 6), although neoatherosclerosis is still a potential limitation of such approach. In a recent study, Osborn et al. (82) have demonstrated that plaque inflammation is an independent predictor of plaque progression, using atheroma rabbit model and serial NIRF-OCT. These preclinical studies will provide an outline for clinical studies once a NIRF catheter is approved.

## Conclusion and Future Directions

Intravascular NIRF molecular imaging offers a new dimension for plaque assessment based on pathobiology, a key driver of coronary events, and is on the verge of clinical translation to human coronary arteries. We envision that intravascular NIRF-OCT or NIRF-IVUS molecular-structural imaging will be used to comprehensively assess the coronary artery of patients already undergoing percutaneous coronary intervention. As PCI is now routinely being performed with intravascular imaging (IVUS or OCT), we anticipate that the only difference for NIRF-OCT or NIRF-IVUS will be the administration of a targeted molecular imaging agent at the start of PCI. Following PCI, completion NIRF-OCT or NIRF-IVUS will be performed, allowing assessment of non-culprit coronary artery segments proximal and distal to the target lesion that was stented. This will allow the generation of an integrated molecular-structural atheroma score reflecting the degree of pathobiology for the culprit artery. In addition another artery could be potentially imaged with the same goal. Ultimately, a NIRF-based pathobiology score will identify high-risk lesions, arteries, and patients, allowing the ability to more precisely target newer atheroma medical therapies (e.g., PCSK9 inhibitors, icosapent ethyl, ezetimibe, GLP1-receptor antagonists, SGLT2 inhibitors) to those at highest risk. Overall, integration of NIRF pathobiology assessment will enable personalized medical therapy for patients with CAD, instead of a one size fits all approach as currently practiced worldwide (e.g., statin and dual anti-platelet therapy, regardless of underlying pathobiological risk).

## Author Contributions

Both authors contributed to the writing of this review article.

## Conflict of Interest

FAJ has received sponsored research grants from Canon and Siemens; he is a consultant for Boston Scientific, Abbott Vascular, Siemens, Acrostak, and Biotronik. Massachusetts General Hospital has a patent licensing arrangement with Canon, and FAJ has the right to receive royalties. The remaining author declares that the research was conducted in the absence of any commercial or financial relationships that could be construed as a potential conflict of interest.
